# Transcriptome analysis reveals that the multiple metabolic pathways were related to gluten polymerization in different quality wheats (Triticum aestivum L.)

**DOI:** 10.1002/fsn3.1769

**Published:** 2020-07-11

**Authors:** Qi Wang, Feng Jia, Xia Zhang, Xiaohua Wang, Jinhe Li, Jinshui Wang

**Affiliations:** ^1^ College of Bioengineering Henan University of Technology Zhengzhou China

**Keywords:** gluten polymerization, RNA sequence, transcriptome, wheat

## Abstract

The rapid development of transcriptome sequencing technology has contributed to the discovery of numerous genes in plant; however, the role of gene expression in postharvest wheat remains largely unexplored. In this study, differentially expressed genes (DEGs) were identified by RNA‐seq in different quality wheats. The 102.6 Gb clean reads had been yielded from the nine RNA‐seq libraries. Typically, there were 1791 upregulated and 2,677 downregulated DEGs, respectively, in strong‐gluten wheat compared with weak‐gluten wheat. Specifically, a total of 4,468 DEGs were classified into 286 Gene Ontology (GO) terms and 131 Kyoto Encyclopedia of Genes and Genomes terms (KEGG). Moreover, the storage protein components, starch and sucrose metabolism, and plant hormone signal transduction‐related genes were discovered, which had involved 109 DEGs. The wet gluten proteins content was 35.24% and 17.36%, and the glutenin macropolymer content was 6.38% and 5.01% between the strong‐ and weak‐gluten wheat, respectively. The POD activities of the different quality wheats were 6,571.14, 5,341.24, and 4,851.48 U/g/min, respectively. The significant difference of starch and sucrose metabolism, hormone, POD, and CAT enzyme along with the higher ATPase activity might potentially affect gluten polymerization, which might thereby result in the different qualities of wheats.

## Graphical abstract

This work firstly subjected transcriptome to reveal the effect of the different quality wheat, and we found that the formation of different quality wheats may be due to the difference of GMP, storage protein, hormone, protein isomerase, antioxidase activity, and energy metabolism.

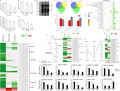

## 1 INTRODUCTION

Wheat flour has extensive applications, including bread, pasta, noodles, dumplings, Chinese steamed bread, pastries, and cookies (Li, Li, Qua, & Wang, 2017; Li, Li, & Bian, 2016). The product attributes are determined by the composition of gluten, which depending on the ingredient formulations and processing conditions, then achieve a required product (Jazaeri et al., 2015). For example, strong‐gluten wheat is preferred for pasta and bread, because its gluten network has good elasticity and ductility (Kumar, Elias, et al., 2013), while medium‐gluten wheat is suitable for making noodles, Chinese steamed bread, and dumplings (Li et al., 2017), and weak‐gluten wheat is only applicable for making cakes, cookies, and pastries. The composition of quality characters among the three wheats with different qualities is shown in Table 1. Nevertheless, strong‐gluten wheat as the model system had been discussed by Bock and Seetharaman (Bock & Seetharaman, 2012). The glutenin subunit has contained the disulfide bonds, which can be linked together into the stable polymers (Tamás et al., 2002). According to polymer theory, glutenin macropolymer (GMP) plays a certain role in the establishment of the gluten network structure, but it can only be achieved in a certain size of glutenin polymers (Veraverbeke & Delcour, 2002). Typically, the effect of GMP on the gluten network is dependent on the wheat gluten composition, as well as grain size distribution and structure (Goesaert et al., 2005), among which the wheat gluten composition refers to the type and proportion of high‐molecular‐weight gene subunit (HMW‐GS) and low‐molecular‐weight gene subunit (LMW‐GS). Besides, the number of cysteine residues in HMW‐GS and LMW‐GS is also a vital factor affecting the GMP content and particle size formation (Yue et al., 2007). Furthermore, the research status on this topic to date has been well summarized in some recent reviews (Bonomi, Iametti, Mamone, & Ferranti, 2013; Delcour et al., 2012; Jazaeri et al., 2015). Nonetheless, the study on the difference among the different quality wheats at transcriptome level has not been reported yet.

**Table 1:** Analysis of the comparison characters among different quality wheat

Variety	Protein content (%)	Wet gluten content (%)	Water absorption (%)	Sedimentation Value (mL)	Stability time (min)	Degree of softening
Xinmai 26	16.04	32.3	65.6	70.9	38.4	31.45 F.U
Fanmai 8	15.42	27.9	53.4	73.5	10.4	68 F.U.
Yangmai 15	10.24	19.7	54.1	23.1	1.1	

## 2 MATERIALS AND METHODS

### 2.1 Materials

Strong‐gluten wheat (Strong, Xinmai 26), medium‐gluten wheat (Medium, Fanmai 8), and weak‐gluten (Weak, Yangmai 15) wheat seeds were used in this study, and all the wheat seeds were planted under natural conditions at the Yellow River farm (Zhoukou, Henan province, China). The seeds were new harvested and dried naturally for about one week, and the seeds size were manually selected. All the wheat seeds were immediately stored in a −80 ℃ freezer after freezed in liquid nitrogen for 20 min, and 0.5 g of seeds was subjected to RNA isolation.

### 2.2 RNA library construction and sequencing

Total RNA extraction using Total RNA Purification Kit (TRK1001, LC Science, Houston TX) following the instructions. The RNA reverse transcription according to the manufacturer's procedure for the mRNA‐Seq sample preparation kit (Illumina, San Diego, USA), the average fragment was 450 bp ( ±50 bp) of the paired‐end libraries, using Illumina Hiseq 4,000 at the following of the merchant's instruction (lc‐bio, China). We used HISAT package (Pertea, Kim, Pertea, Leek, & Salzberg, 2016), which initially remove a portion of the reads based on quality information accompanying each read and then maps the reads to the reference genome. HISAT2 allows multiple alignments per read (up to 20 by default) and a maximum of two mismatches when mapping the reads to the wheat reference (Appels et al., 2018). HISAT2 builds a database of potential splice junctions and confirms these by comparing the previously unmapped reads against the database of putative junctions (Pertea et al., 2016). We used the Cutadapt software to remove the adaptors (Martin, 2011). The transcripts were spliced and merged with StringTie 1.3.0 (Pertea et al., 2016). The differentially expressed genes (DEGs) were selected by R package edgeR with a threshold of the *p*‐value < .05 and fold change >2 or fold change <−2 (Robinson, McCarthy, & Smyth, 2010). The GO database (http://geneontology.org) was used to analyze GO terms enrichment of DEGs, and the KEGG database (http://www.kegg.jp/kegg) was used to identify the significantly enriched metabolic pathways. According to the functional difference, cluster analysis was used to determine the expression patterns of DEGs on different wheat varieties based on the FPKM (expected number of Fragments Per Kilobase of transcript sequence per Millions base pairs sequenced) values (Trapnell et al., 2010).

### 2.3 Measurements of antioxidant enzyme activity

Analysis of superoxide dismutase (POD) and catalase (CAT) activities were performed as previously procedure (Wan et al., 2009).

### 2.4 Preparation GMP and SDS‐PAGE

The GMP isolation from flour was performed as previously described (Yue et al., 2019), with some modifications. 0.5 g flour was powdered again in liquid N_2_ rapidly and suspended in 6 ml of 1.5% SDS, and vortex at room temperature for 2 hr, then centrifuged at 4 ℃ for 20 min at 16,000 g, once again. The pellet of GMP that forms a transparent gel layer. 1.5 ug of GMP proteins was conducted to SDS‐PAGE. The gels were stained with 0.1% CBB R‐250 overnight and destained in methanol/acetic acid (1:3) until a clear background. Protein gels were photographed using a scanner GS‐900TM Calibrated Densitometer (Bio‐RAD).

### 2.5 Analysis of gluten properties

For flour samples, gluten properties were analyses following the Brabender manufacturer's procedure. Tests were done in duplicate, and standard deviations are displayed as error bars.

### 2.6 Real‐time fluorescent quantitative PCR analysis

The total RNA extraction using a RNA prep pure Kit (BIOFIT, Chengdu, China) and subsequently for qRT‐PCR analysis. Following the reagent instructions, the reverse transcription was used a TUREscript 1st Stand cDNA SYNTHESIS Kit (Aidlab, Beijing, China) for 1.0 μg RNA. The qRT‐PCR assay was processed in an analytikjena‐qTOWER2.2 (Germany) using SYBR Green Mix (Bio‐Rad, CA, USA). The internal control was used 18S rRNA gene. All primers and their sequences are listed in Table S1.

## 3 RESULTS

### 3.1 Analysis of the properties in different quality wheats

**Figure 1:**
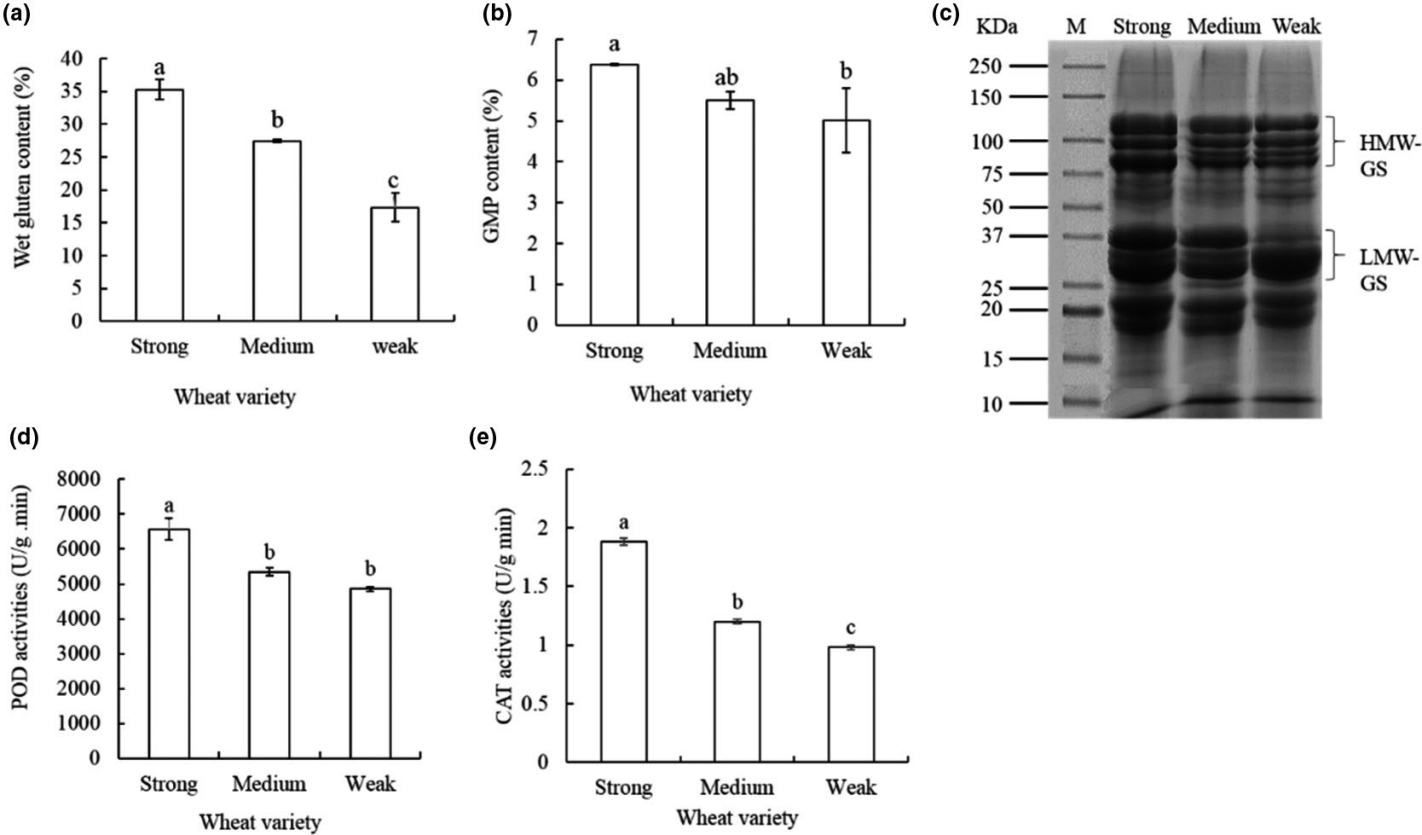
(a) Wet gluten content, 35.24%, 27.47, and 17.36% were observed in the strong‐gluten wheat, the medium‐gluten wheat, and the weak‐gluten wheat, respectively. (b) GMP content, the content of GMP was 6.38% and 5.01% in the strong‐gluten wheat and the weak‐gluten wheat, respectively. (c) The composition of GMP between the strong‐, medium‐, and weak‐gluten wheat. d POD activities, The POD activities of the strong‐gluten wheat, medium‐gluten, and weak‐gluten wheat were 6,571.14, 5,341.24, and 4,851.48 U/g/min, respectively. e CAT activities, CAT activities among the three different gluten wheat were 1.88, 1.2, and 0.98 U/g/min, respectively. Means of three repeats and standard errors are presented; the same letter above the column indicates no significant difference at *p* < .05

The highest wet gluten content of 35.24% was observed in the strong‐gluten wheat, followed by the medium‐gluten wheat of 27.47, while the lowest wet gluten content in the weak‐gluten wheat was 17.36% (Figure 1a). The GMP content was higher (6.38%) in the strong‐gluten wheat than the weak‐gluten wheat (5.01%) (Figure 1b). The peak maximum time (PMT) and Torque maximum (BEM) reflected the protein content in wheat, and significantly different was observed between the strong‐gluten and weak‐gluten wheat (Table 2). Torque before maximum (AM) and A3 reflected the stretching area of flour, and shows a negative correlation with the gluten strength (Table 2). The composition of GMP in flour was included with HMW‐GS, which was linked LMW‐GS through the intermolecular disulfide bonds. The HMW‐GS of strong‐gluten wheat was higher than that of weak‐gluten wheat, and the numbers of HMW‐GS band in strong‐gluten wheat were lesser than that in weak‐gluten wheat (Figure 1c). Nevertheless, there was no obvious difference of the content of salt‐soluble and water‐soluble protein between the strong‐gluten, medium‐gluten, and weak‐gluten wheat in Figure S2.

**Table 2:** Changes of Glutopeak parameters among the different quality wheat

	PMT	BEM	AM	PM	A3
Strong	86 ± 2.1254*	74 ± 6.8724*	41 ± 4.2325*	51 ± 5.6322*	841 ± 19.5134*
Medium	78 ± 1.9658	51 ± 6.2422	38 ± 2.8563*	40 ± 4.2214	586 ± 25.3422
Weak	55 ± 4.4554	47 ± 3.4321	23 ± 2.2551	35 ± 4.0312	528 ± 15.2311

Note

Means of three independent samples and standard errors are presented; the asterisk indicates significant difference at *p* < .05.

### 3.2 Comparison of antioxidant enzyme activity

The peroxidase (POD) activities of the strong‐gluten wheat, medium‐gluten wheat, and weak‐gluten wheat were found 6,571.14, 5,341.24, and 4,851.48 U/g/min, respectively. The POD activity of strong‐gluten wheat was 1.35‐fold higher than that of weak‐gluten wheat, while it was almost the same level between medium‐gluten wheat and weak‐gluten wheat (Figure 1d). There were significant differences of catalase (CAT) activity among the three different gluten wheats, which were 1.88, 1.2, and 0.98 U/g/min, respectively (Figure 1e). The results showed that POD and CAT activity might be associated with the different qualities of wheat.

### 3.3 RNA‐seq sequencing and DEGs analysis

**Figure 2:**
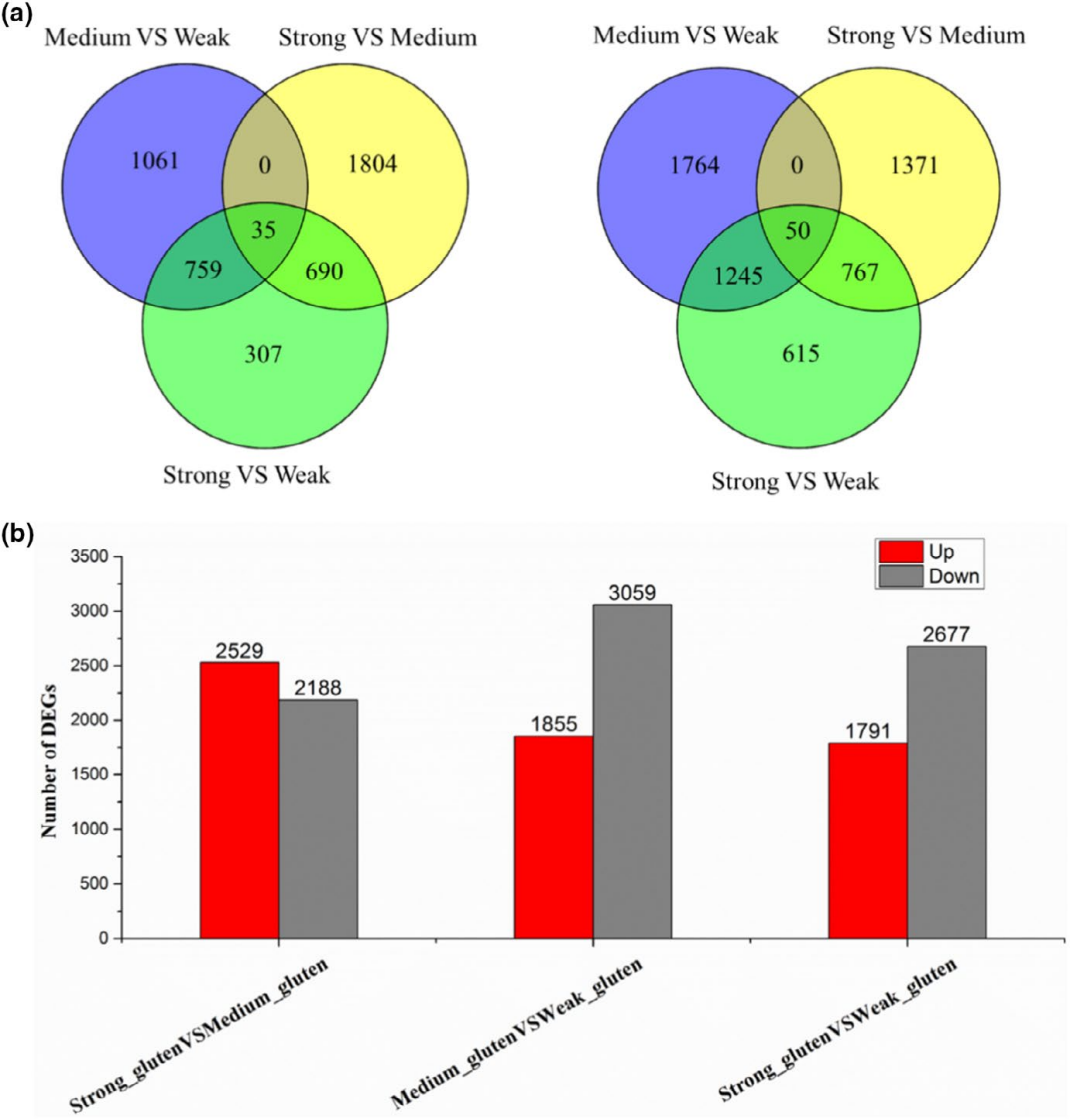
The numbers of unigenes detected in the three quality wheats are displayed in Venn charts. (a) upregulated genes or (b) downregulated genes. Volcanic diagrams indicated the DEGs differentially expressed pattern (c, d), the red dots indicate DEGs with upregulation, and the blue dots indicate that downregulation. Strong, Strong‐gluten wheat; Medium, Medium‐gluten wheat; Weak, Weak‐gluten wheat

**Figure 3:**
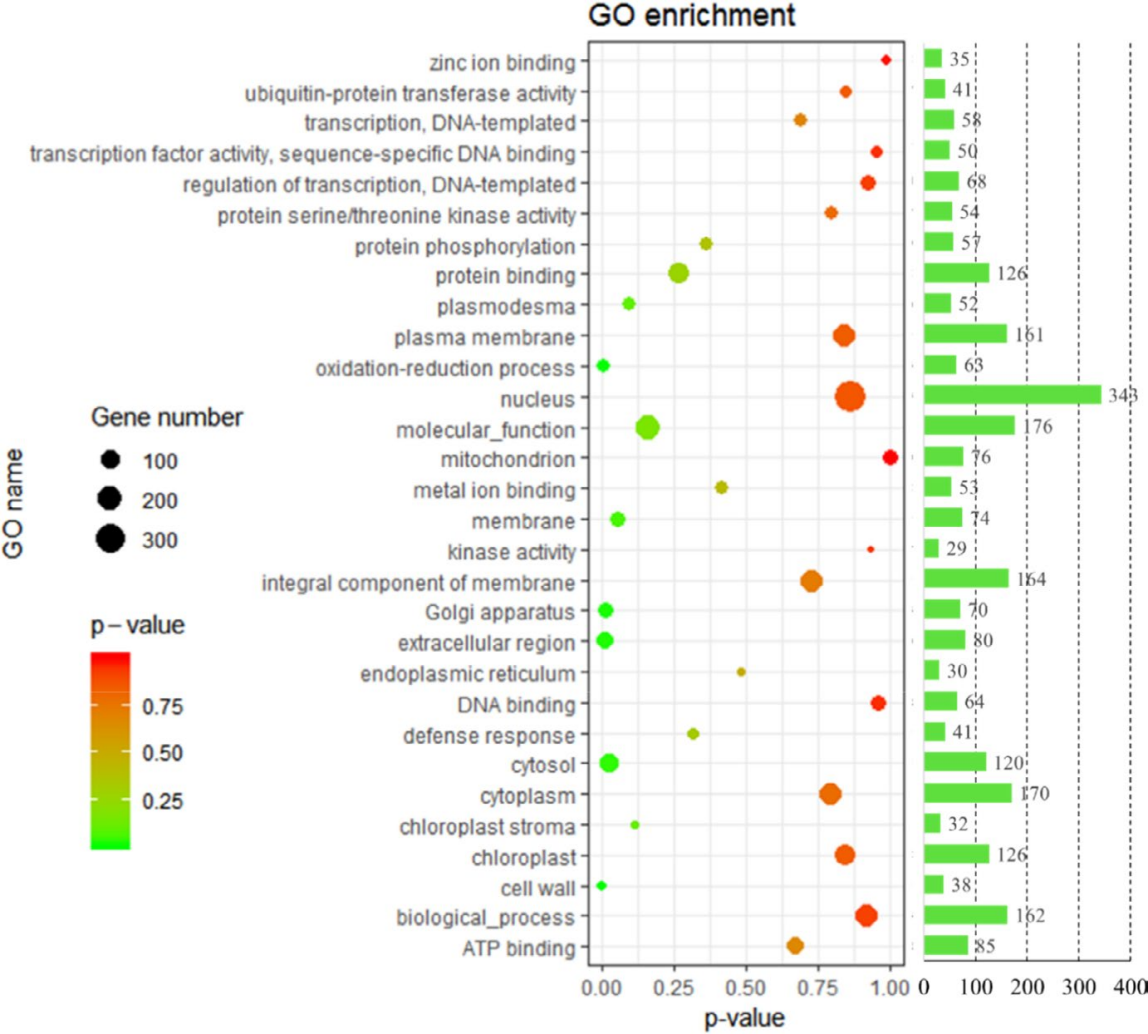
Gene ontology (GO) terms that were significantly enriched in the DEGs between the three kinds of wheat. GO terms related to the gluten polymerization were shown. The numbers of DEGs in each GO term were collected for DEGs (*p* < .05) among the three quality wheats

Nine cDNA libraries (three biological replicates for one sample, respectively) prepared from different quality wheat, and sequencing platform is the Illumina HiSeq 4,000. There were 103.69 Gb raw data were generated, and 102.6 Gb clean reads were obtained from the nine different quality wheat libraries by removing adapters and low quality sequences (1, reads containing sequencing adaptors; 2, reads containing sequencing primer; 3, nucleotide with q quality score lower than 20) (Table 3). For comparative transcriptome analysis in different quality wheat seeds, the transcriptome raw data of wheat were uploaded to Gene Expression Omnibus (GEO) (GEO number: GSE122699). Finally, 4,914, 4,717, and 4,468 DEGs were obtained from the medium‐gluten versus weak‐gluten, strong‐gluten versus medium‐gluten, and strong‐gluten versus weak‐gluten wheat transcriptome libraries, respectively. All assemble DEGs were first searched against the GO and KEGG databases using the DIAMOND (Buchfink, Xie, & Huson, 2015) software with threshold of E‐value ≤1E‐5. In total, 2,529 DEGs were upexpressed and 2,188 DEGs were downexpressed between strong‐gluten wheat and medium‐gluten wheat. There were 4,914 DEGs between medium‐gluten wheat and weak‐gluten wheat; meanwhile, 1791 DEGs were upexpressed and 2,677 DEGs were downexpressed in strong‐gluten wheat than weak‐gluten wheat. Differentially expressed genes in the three compared groups were shown in Venn diagram (Figure 2), which was related to the quality of wheat. Such as HMW‐GS, LMW‐GS, gliadin, and globulin genes were upregulation in the strong‐gluten and medium‐gluten wheat, and downregulation in weak‐gluten wheat. E3 ubiquitin‐protein ligase and UDP‐glucose 4‐epimerase were upregulated in the weak‐gluten wheat, and downregulated in the strong‐gluten and medium‐gluten wheat. The biological process category has the largest number of DEGs among the three GO terms, the second was the molecular function category, and cellular component category was the least among the different quality wheat. Within the cellular component category, the nucleus, cytoplasm, and integral component of membrane have clustered the highest number of DEGs. Meanwhile, regulation of transcription and oxidation–reduction process were enriched in the biological process category. Protein binding and ATP binding take the largest proportion in the molecular function category (Figure 3; Table S2). There were 730 DEGs assigned to 123 KEGG pathways. The largest pathway was plant–pathogen interaction (71 genes, 9.73%), followed by starch and sucrose metabolism (49 genes, 6.71%). The detailed results are shown in Table S3.

**Table 3:** Summary of assembly and annotation of different quality wheat transcriptome

Samples	Raw reads	Valid reads	Mapped reads	Valid Ratio (reads)	Q20%	Q30%	GC content%
Strong	76,419,664	75,658,012	70,079,989	99.01	99.25	94.99	56.50
Medium	76,580,480	75,675,033	71,372,833	98.81	99.50	97.24	56.50
Weak	77,430,846	76,670,792	71,670,030	99.02	99.45	96.73	56.50

### 3.4 Identification of quality‐associated genes

**Figure 4:**
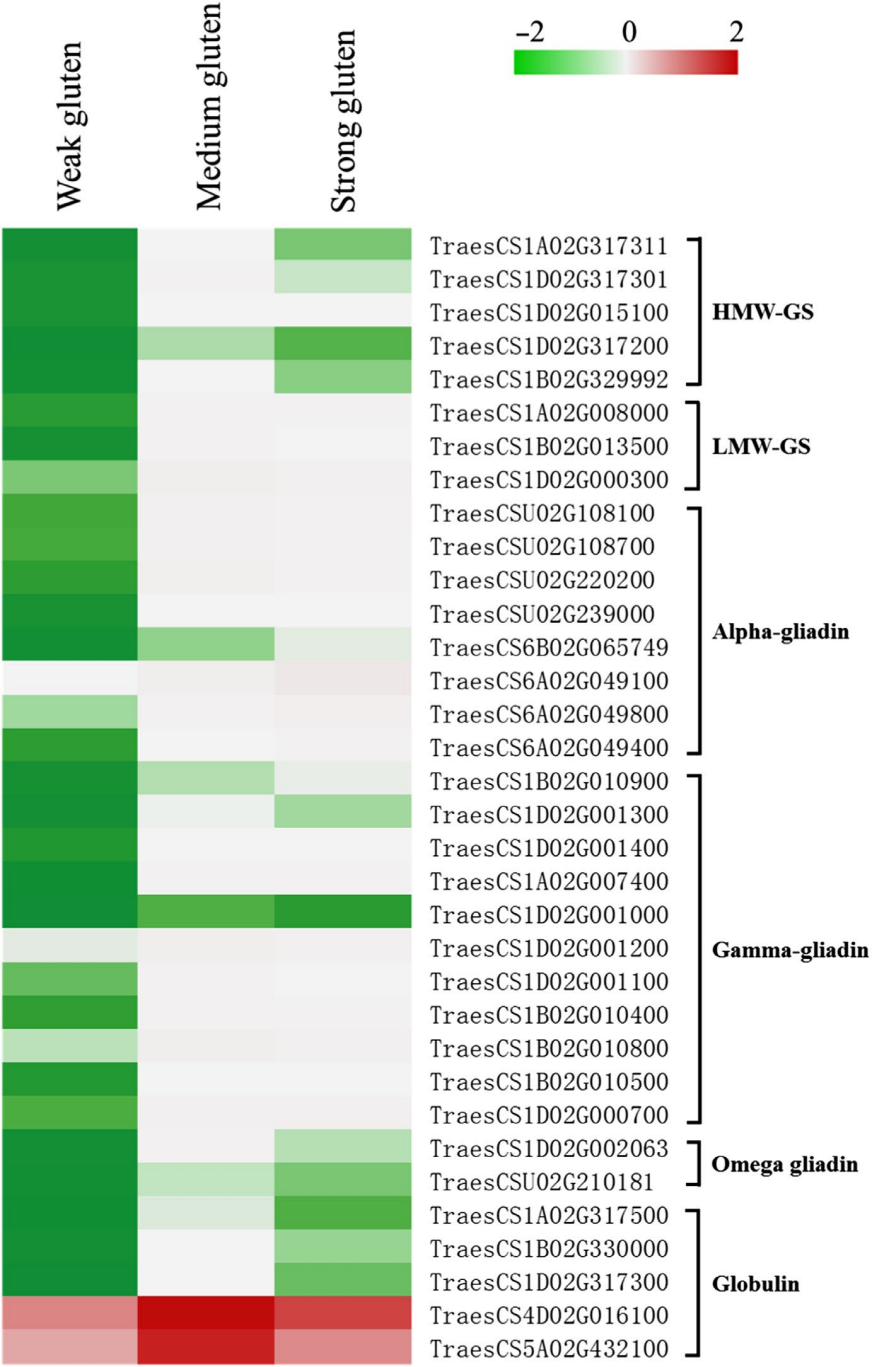
Expression pattern of DEGs from the three varieties wheat of transcriptome comparisons. The FPKM of genes related to gluten polymerization in three samples was scaled together using an in‐house Perl program and is represented in a clustering heatmap by R. Green to red colors reflect gene expression levels indicated as log 10(FPKM + 1) (value of −2–2). A detailed heatmap with DEGs IDs and annotations was included (Table S6). Abbreviations: HMW‐GS, high‐molecular‐weight gene subunit; LMW‐GS, low‐molecular‐weight gene subunit

GO enrichment analysis of DEGs shows that regulation of transcription, oxidation–reduction process, and protein phosphorylation were enriched GO term, and defense response, secondary metabolite biosynthetic process, and protein folding were take possession of a considerable proportion (Figure 3). Moreover, through the pathway analysis, four pathways related to the gluten polymerization: starch and sucrose metabolism, plant hormone signal transduction, protein processing in endoplasmic reticulum, and phenylpropanoid biosynthesis were discovered and identified (Figure S1, *p*‐values ≤ 0.05). The 4 HMW‐GS and 7 LMW‐GS genes were upregulated in strong‐gluten wheat and medium‐gluten wheat than in the weak‐gluten wheat. A large number of gliadin proteins, including α‐, γ‐, and ω‐gliadin, were upregulated in strong‐gluten wheat than in the weak‐gluten wheat (Figure 4). A total of 4 and 5 DEGs were identified for the protein isomerase and POD enzyme, in the strong‐gluten wheat and weak‐gluten wheat, respectively (Table S7).

### 3.5 The changes of four storage protein gene expression among the different quality wheat

Gluten and gliadin proteins are the two most important storage proteins that determine the quality of wheat flour. The expression of the HMW‐GS (high molecular weight gene subunit) and LMW‐GS (high molecular weight gene subunit) of gluten was significantly higher in the strong‐gluten and medium‐gluten wheat than in the weak‐gluten wheat. Alpha‐, beta‐, and omega‐gliadin had the same expression tendency. The globulin expression was the highest in the medium‐gluten wheat than the other quality wheat. The results suggest that the difference among the different quality of wheat was mainly manifested in the gene expression of stored protein (Figure 4).

### 3.6 Different quality wheat transcriptional changes of starch and sucrose metabolism‐related genes

**Figure 5:**
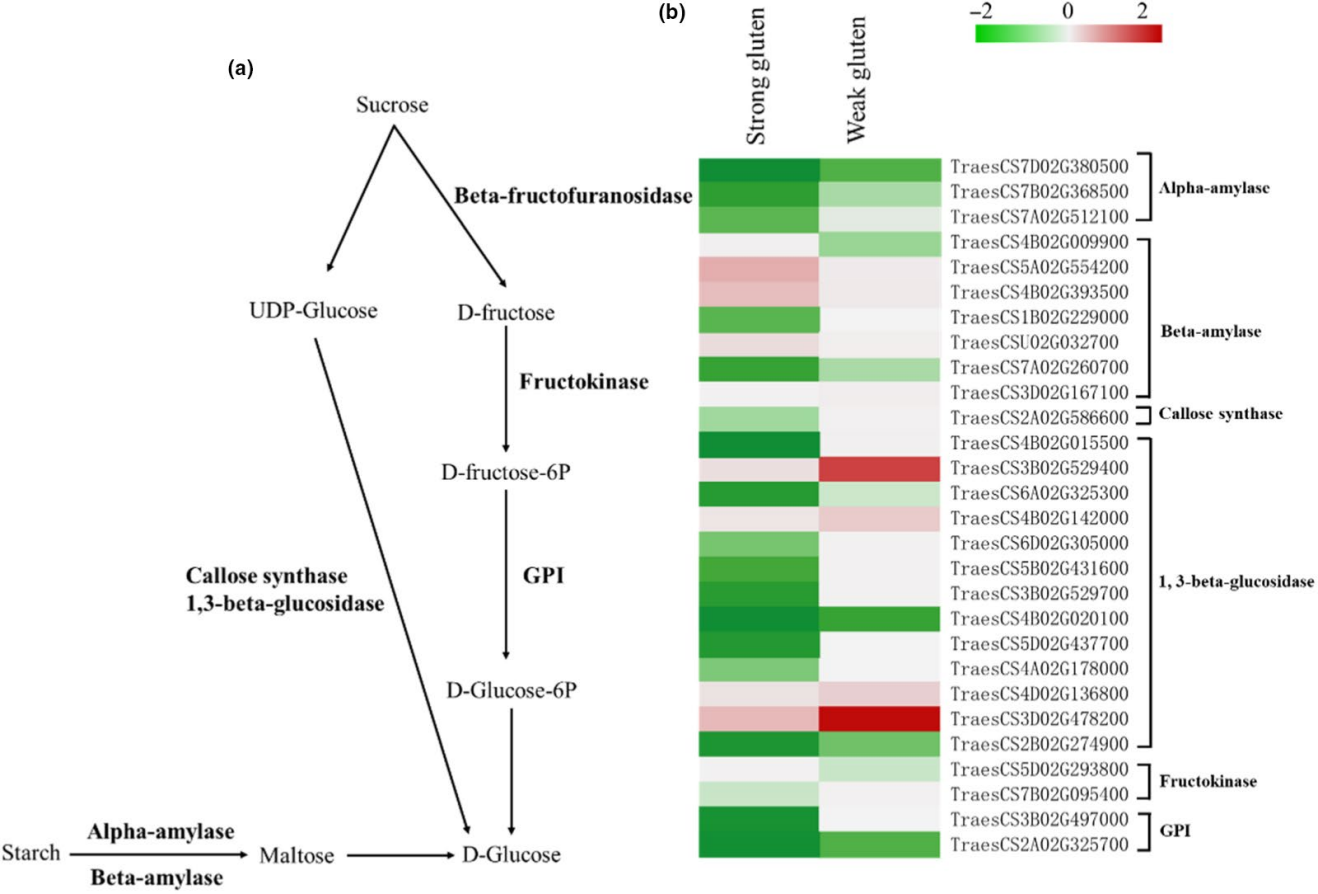
Changes in expression of starch and sucrose metabolism genes between the strong‐gluten and weak‐gluten wheat. Simplified starch and sucrose metabolic pathway in plants (A). Fold changes (log_2_ scale) in the expression of starch and sucrose metabolism genes between strong‐gluten wheat and weak‐gluten wheat. The log_2_ fold change values are shown by the negative and positive numbers on the bar, and the color scale shows upregulation (red) and downregulation (green) of the respective genes. Fold changes (linear‐scale) in expression and the associated P‐values are presented in Table S2. Abbreviations: GPI, galacturan 1,4‐alpha‐galacturonidase.

There were 34 genes annotated as starch and sucrose metabolism are found in the wheat genome, including 4 alpha‐amylase and 7 beta‐amylase that an enzyme catalyzing starch to maltose, 3 callose synthase, and 14 1, 3‐beta‐glucosidase catalyzing UDP‐Glucose to glucose, 1 beta‐fructofuranosidase catalyzing sucrose to D‐fructose, 3 fructokinase catalyzing D‐fructose D‐fructose‐6P, and, finally, 3 glucose‐6‐phosphate isomerase (GPI) catalyzing D‐fructose‐6P to D‐glucose. All of these genes exhibited differently expression among the three quality wheat. Our results suggest that the decomposition in weak‐gluten wheat is higher than that in strong‐gluten wheat (Figure 5, Table S4).

### 3.7 Transcriptional alteration of specific hormone‐related genes among the different quality wheat

**Figure 6:**
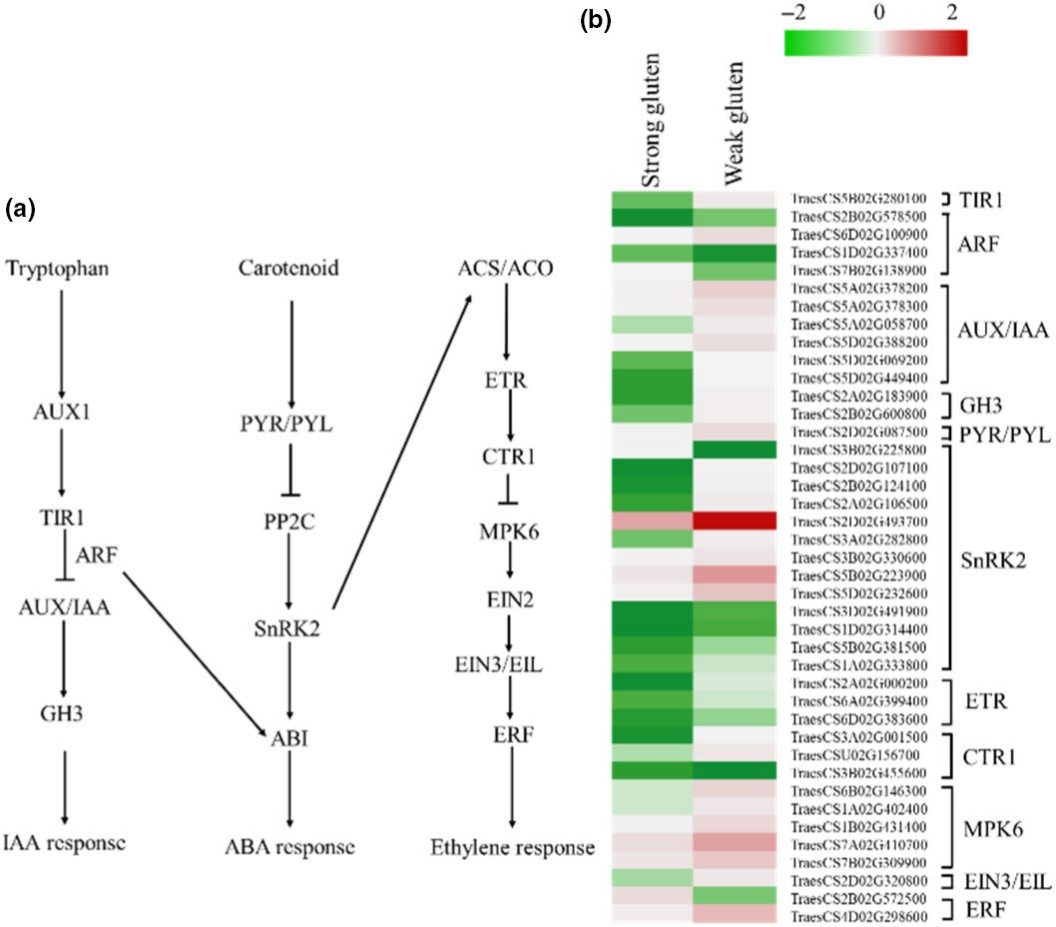
Changes in expression of hormone signaling genes between the strong‐gluten and weak‐gluten wheat. Simplified hormone in plants (A). Fold changes (log_2_‐scale) in the expression of starch and sucrose metabolism genes between strong‐gluten wheat and weak‐gluten wheat. The log_2_ fold change values are shown by the negative and positive numbers on the bar, and the color scale shows upregulation (red) and downregulation (green) of the respective genes. Fold changes (linear‐scale) in expression and the associated P‐values are presented in Table S2. Abbreviations: TIR1, Transport inhibitor response 1; ARF, auxin response factor; AUX/IAA, auxin‐responsive protein; GH3, Gretchen Hagen3; PYR/PYL, pyrabactin resistance/pyr1‐Like; PP2C, 2C type protein phosphatases; SnRK2, sucrose nonfermenting 1‐related protein kinases 2; ETR, ethylene receptor; CTR1, constitutive triple response 1; MPK6, mitogen‐activated protein kinase 6; EIN3, ethylene‐insensitive protein 3; ERF, ethylene‐responsive transcription factor

Transport inhibitor response 1 (TIR1), 3 auxin response factor (ARF), 6 auxin/IAA‐responsive protein (AUX/IAA), and Gretchen Hagen 3 (GH3) belong to auxin signal pathway, and upregulated in weak‐gluten wheat than in strong‐gluten wheat. PYRABACTIN RESISTANCE and PYR1‐Like (PYR/PYL) is a receptor of ABA (Abscisic acid), which suppressed the ABA signal factor phosphatase type 2C (PP2C). The PP2C increases the expression of SNF1‐related kinase 2 (SnRK2) (a receptor of ABA). Interestingly, the PYR/PYL, PP2C, and SnRK2 were upregulation in weak‐gluten wheat than in the strong‐gluten wheat. Ethylene response (ETR) and constitutive triple response (CTR1) as ethylene receptor were located in the endoplasmic reticulum, and upregulated in weak‐gluten wheat and downregulated in strong‐gluten wheat. 5 MPK6 (mitogen‐activated protein kinase 6), 1 EIN3 (ethylene‐insensitive protein 3), and 2 ERF (ethylene‐responsive transcription factor) genes exhibited upregulation in weak‐gluten wheat relative to strong‐gluten wheat (Figure 6; Table S5).

### 3.8 Validation the transcriptomic sequencing results by qRT‐PCR

**Figure 7:**
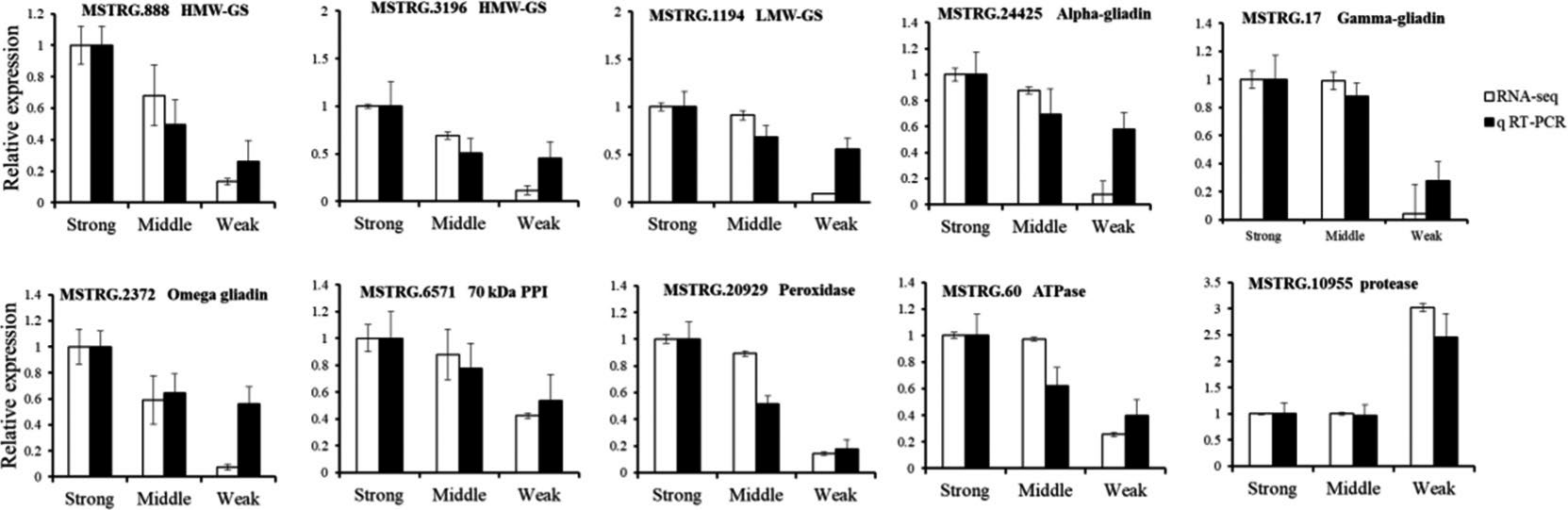
qRT‐PCR validation of DEGs identified by RNA‐Seq. Ten DEGs including nine upregulated genes and one downregulated genes from the three varieties wheat of transcriptome comparisons (strong‐gluten vs medium‐gluten vs weak‐gluten wheat) were randomly selected for qRT‐PCR confirmation. The white and black column represent RNA‐Seq and qRT‐PCR data, respectively. The wheat 18S RNA gene was used as an internal control, and these experiments were repeated with three biological samples. Error bars indicate the standard errors. Abbreviations: HMW‐GS, high‐molecular‐weight gene subunit; LMW‐GS, low‐molecular‐weight gene subunit; PPI, peptidyl‐prolyl isomerase; ATPase, adenosine triphosphatase

We applied real‐time fluorescent quantitative PCR (qRT‐PCR) assays to verify the RNA‐Seq data, and the template using was the wheat seeds used for RNA‐Seq analysis (one of three duplicated samples RNA), randomly select 10 genes for validation, including 9 downregulated genes and one upregulated genes from the different quality wheat (Table S6). The expression patterns of the 10 genes were similar between the qRT‐PCR results and the RNA‐Seq, which indicated that the RNA‐Seq results were highly reliable (Figure 7).

## 4 DISCUSSION

The transcriptome data revealed that the HMW‐GS expression and LMW‐GS expression were upregulated in the strong‐ and medium‐gluten than in the weak‐gluten wheat. Meanwhile, compared with the weak‐gluten wheat, numerous peptidyl‐prolyl isomerase genes in strong‐gluten wheat had been upregulated, which might promote the cross‐linking of low‐molecular‐weight proteins to high‐molecular‐weight proteins (Don, Mann, Bekes, & Hamer, 2006). Tyrosine kinase was regulated by PPIse, which could further cross‐link with different protein containing the lysyl, tyrosyl, and cysteinyl residues (Selinheimo, Lampila, Mattinen, & Buchert, 2008). Specifically, proline residues in the homologous protein are highly conserved, which had revealed the potential role of proline in protein recognition and function, suggesting that HMW‐GS might be folded by PPIases due to its high proline (10 mol %) content (Kumar, Khatkar, & Kaushik, 2013). Peroxidase generally exists in various kinds of wheats, which could catalyze hydrogen peroxide into water (Every, Simmons, & Ross, 2006) and can provide a donor molecule associated with oxidation (Lai, Wang, Chang, & Wang, 2006). To be specific, add horseradish POX/H_2_O_2_ or catechol into poor baking quality, the flour quality can be improved, and the quality of doughs and breads made from the above‐mentioned flours have been improved (Kieffer, Matheis, Hofmann, & Belitz, 1981). In addition, POX can also improve dough tolerance to over‐mixing in the meantime of slightly increasing the final loaf volume (Dunnewind, Vliet, & Orsel, 2002; Gélinas, Poitras, Mckinnon, & Morin, 1998). Such results indicate that peroxidases affect the gluten network through two modes, one is the gluten proteins cross‐linking, and the other attached arabinoxylans to gluten proteins (Buchert et al., 2010). Research shows that approximately 25% proteins of the formed gel that could be cleavage by proteolytic (Neukom & Markwalder, 1978). In addition, H_2_O_2_ could affect the viscosity of flour fraction in the existence of native POX (Hoseney & Faubion, 1981). Similar to POX, Catalase can also catalyze the conversion reaction, where tyrosine acts as the hydrogen donor. Therefore, CAT can also affect protein polymerization (Honold & Stahmann, 1968). Finally, leading to higher protein polymerization levels of strong‐ and medium‐gluten wheats. However, research on CAT related to gluten protein polymerization has rarely been carried out (Brijs et al., 2009; Joye, Lagrain, & Delcour, 2009).

Moreover, reports from barley, maize, and legumes have indicated that ATP levels are correlated with the storage‐related processes, and the storage product might be divided into different category according to the energy state (Borisjuk et al., 2010; Einali & Valizadeh, 2017; Rolletschek, Koch, Wobus, & Borisjuk, 2010; Rolletschek, Weschke, Weber, Wobus, & Borisjuk, 2004). ATP is mainly generated from the process of biological oxidation, including carbohydrate metabolism, fatty acid degradation, and amino acid metabolism, and energy generated from the above metabolism could also be used by other biological activities of seeds in protein synthesis or polymerization. The increase in proteolysis and gluconeogenesis activities is associated with the Lipid metabolism, which will lead to the free amino acids and carbohydrates accumulation (Einali & Sadeghipour, 2007; Nezamdoost, Tamaskani, Abdolzadeh, & Sadeghipour, 2009); typically, the free amino acids will be used for protein synthesis. Likewise, carbohydrate accumulation also plays a crucial role in seed dormancy breaking (Li & Ross, 1990). Recently, research indicates that a second metabolic switch has found from transition period to the desiccation phase (Fait et al., 2006), and seed primarily changed the metabolism pattern from oil and storage protein accumulation to the contents of free amino acids and sugars increasing, while fatty acids degradation could provide the energy for the desiccation seed (Chia, Pike, & Rawsthorne, 2005).

## 5 CONCLUSIONS

In summary, this is the first study investigating the effect of gene expression profiles in different quality wheats. It is proposed that the different qualities among the three kinds of wheats can be ascribed to multiple factors, including storage protein gene expression, hormone signal transduction, protein isomerase, antioxidase activity, and energy metabolism. In strong‐gluten wheat, the content of HMW‐GS and LMW‐GS is higher than that in weak‐gluten wheat, and the activities of cysteine synthase and isomerase are also enhanced, which may promote the cross‐linking of low‐molecular‐weight protein to high‐molecular‐weight protein. Meanwhile, POD enzyme can strengthen the gluten network and CAT enzyme will affect gluten polymerization, along with higher ATPase activity, which can provide energy for protein polymerization reaction, meanwhile in consideration of the wheat varietal differences, eventually leading to significant differences among the different quality wheats.

## CONFLICT OF INTEREST

The authors declare that they have no conflicts of interest with the contents of this article. This study does not involve any human testing.

## AUTHOR CONTRIBUTIONS

Jinshui Wang conceived the idea, designed the experiments, and precisely revised the manuscript. Qi Wang analyzed the data and prepared the manuscript. Feng Jia helped in data interpretation and software using. Xia Zhang analyzed the data and carry out qRT‐PCR analysis. Xiaohua Wang and Jinhe Li collected plant materials. All authors read and approved the final manuscript.

## Supporting information

Supplementary Material

Supplementary Material

Supplementary Material

Supplementary Material

Supplementary Material

Supplementary Material

Supplementary Material
